# Medicina de Precisão Populacional em Cardiologia: Reduzindo Lacunas em Saúde por Meio da Estratificação Integrada de Risco

**DOI:** 10.36660/abc.20260546

**Published:** 2026-07-31

**Authors:** Leonardo Pinto de Carvalho, Mark Y. Chan

**Affiliations:** 1 Escola Paulista de Medicina Universidade Federal de São Paulo São Paulo SP Brasil Disciplina de Cardiologia – Escola Paulista de Medicina – Universidade Federal de São Paulo, São Paulo, SP – Brasil; 2 Dante Pazzanese Institute of Cardiology São Paulo SP Brasil Dante Pazzanese Institute of Cardiology (IDPC), São Paulo, SP – Brasil; 3 Departamento de Cardiologia Yong Loo Lin School of Medicine National University of Singapore Cingapura Departamento de Cardiologia – Yong Loo Lin School of Medicine – National University of Singapore – Cingapura; 4 Cardiovascular Disease National Collaborative Enterprise Rochester Park Cingapura Cardiovascular Disease National Collaborative Enterprise (CADENCE), Rochester Park – Cingapura

**Keywords:** Medicina de Precisão Populacional, Estratificação Integrada de Risco, Cardiologia

## Introdução

O paradigma da medicina cardiovascular está passando por uma transformação profunda. Durante décadas, o alicerce da cardiologia baseada em evidências foi a aplicação dos resultados de grandes ensaios clínicos randomizados aos pacientes individuais. Embora essa abordagem de “tamanho único” tenha, sem dúvida, reduzido a morbidade e a mortalidade cardiovascular em escala global, ela pressupõe que o paciente “médio” representado nos ensaios clínicos reflete com precisão o indivíduo sentado na sala de consulta. Na prática, porém, os clínicos continuam enfrentando grande variabilidade entre pacientes, diagnóstico tardio ou impreciso de doenças raras e comuns, e tratamentos desenvolvidos para o paciente médio frequentemente apresentam eficácia limitada quando aplicados a populações diversas.^1^

Estamos agora entrando na era da medicina de precisão, que promete substituir o cuidado impreciso por estratégias orientadas por dados que integram informações populacionais com insights moleculares. Essa abordagem busca gerar planos de tratamento e cuidado direcionados, baseados nas características clínicas, genômicas e de estilo de vida de cada paciente.

Na cardiologia, entretanto, a medicina de precisão deve ir além do sequenciamento genético individual e incorporar a medicina de precisão populacional. Essa perspectiva mais ampla reconhece que fatores étnicos, demográficos, sistêmicos e genéticos influenciam profundamente o risco cardiovascular, os fenótipos das doenças e as respostas aos tratamentos. O uso disseminado de escores de risco convencionais em populações multiétnicas — combinado com evidências emergentes de riscos de mortalidade ocultos em grupos etários específicos, a integração de biomarcadores moleculares dinâmicos e a incorporação de escores de risco poligênicos – destaca a urgência dessa mudança de paradigma.

### Disparidades étnicas e as limitações dos escores de risco convencionais

Um exemplo contundente da necessidade de uma medicina de precisão específica para populações é ilustrado pelos desfechos de longo prazo após o infarto agudo do miocárdio (IAM). Em um estudo que avaliou mais de 15.000 pacientes com IAM entre três grandes grupos étnicos asiáticos (chineses, malaios e indianos) em Singapura, foram identificadas disparidades significativas na mortalidade de longo prazo.^2^ Embora os pacientes chineses tenham apresentado o maior risco basal segundo o escore do *Global Registry of Acute Coronary Events* (GRACE) – com mediana de 144, em comparação com 138 entre malaios e 131 entre indianos — o padrão de mortalidade cardiovascular em 12 anos foi marcadamente diferente.

Os pacientes malaios exibiram as maiores taxas de mortalidade geral e cardiovascular específica em 12 anos (46,2% e 32,0%, respectivamente), seguidos pelos chineses (43,0% e 27,0%) e pelos indianos (35,9% e 25,2%). Essa discordância entre a estratificação de risco basal convencional e os desfechos de longo prazo evidencia uma limitação crítica: escores de risco derivados predominantemente de populações específicas podem não ser aplicáveis com precisão a outras.

Portanto, a medicina de precisão populacional exige a recalibração de modelos de risco estabelecidos para incorporar a heterogeneidade étnica, garantindo que subgrupos de alto risco recebam as intervenções intensificadas de que realmente necessitam.^2^

### Mortalidade oculta em pacientes jovens: Revelando lacunas de dados

Outra lacuna crítica de dados na medicina cardiovascular de precisão diz respeito aos pacientes jovens com IAM. Apesar da crença convencional de que pacientes jovens com IAM apresentam um prognóstico mais favorável do que indivíduos mais velhos, uma análise abrangente de pacientes jovens com IAM em Singapura revelou uma realidade preocupante quando esses pacientes foram comparados com controles da população geral pareados por idade, em vez de pacientes mais velhos com IAM.^3^ Entre 601 pacientes jovens com idade ≤40 anos, as mortalidades por todas as causas e cardiovascular em 12 anos foram de 12,8% e 9,2%, respectivamente – cinco vezes menores do que em pacientes mais velhos. No entanto, quando comparados com uma população de referência pareada por idade utilizando a metodologia do índice de sobrevivência relativa, os pacientes jovens com IAM permaneceram em risco significativamente elevado.

O índice de sobrevivência relativa de cinco anos para pacientes jovens foi de 0,942 (IC 95% 0,918–0,960), indicando que sua sobrevivência foi aproximadamente 5,8% pior do que o esperado para sua faixa etária. Esse achado revela um insight crucial: sobreviventes jovens de IAM estão “passando despercebidos” pelas avaliações de risco convencionais. Eles parecem ter desfechos favoráveis quando comparados apenas com pacientes mais velhos com IAM, mas carregam uma mortalidade excessiva substancial em relação aos seus pares da mesma idade que não sofreram IAM.

Essa discordância representa uma grande lacuna no cuidado cardiovascular – a falha em reconhecer que pacientes jovens com IAM constituem um fenótipo clínico distinto e complexo, que não é capturado com precisão pelos fatores de risco tradicionais. Sua avaliação exige testes moleculares e genéticos especializados para garantir diagnóstico e estratificação de risco adequados.^3^

### Biomarcadores Moleculares e Estratificação Dinâmica de Risco: Dos Fenótipos de Receptores Plaquetários às Assinaturas Lipidômicas

A evolução rumo à medicina de precisão vem sendo cada vez mais sustentada pelos avanços na fenotipagem molecular e nas tecnologias ômicas. Compreender a fase aguda do infarto do miocárdio exige reconhecer a interação dinâmica entre múltiplos sistemas biológicos, começando pelos fenótipos funcionais das respostas mediadas por receptores plaquetários. Notavelmente, mesmo antes de o perfil de microRNAs circulantes emergir como uma ferramenta potencial de precisão, estudos funcionais já haviam demonstrado que a modulação precoce da reatividade plaquetária mediada por receptores durante as síndromes coronárias agudas possui significado prognóstico.

Em um estudo prospectivo envolvendo 928 pacientes consecutivos hospitalizados por síndrome coronariana aguda, testes duplos de reatividade plaquetária revelaram que a alta reatividade plaquetária sob aspirina e a alta reatividade plaquetária sob antagonistas do receptor de ADP estavam independentemente associadas ao aumento de trombose de stent intra-hospitalar e eventos cardiovasculares maiores (MACE).^4^ Importante destacar que a adição das medidas iniciais de alta reatividade plaquetária sob antagonistas do receptor de ADP ao escore GRACE melhorou significativamente a discriminação para MACE em 30 dias (o C-statistic aumentou de 0,649 para 0,803), demonstrando que os fenótipos funcionais dos receptores plaquetários fornecem informação prognóstica incremental além da estratificação clínica convencional. Isso sugere que os fenótipos plaquetários mediados por receptores na fase aguda não são meros epifenômenos, mas determinantes mecanisticamente relevantes do risco trombótico que merecem ser incorporados na medicina de precisão.

Com base nessa fundamentação da biologia funcional das plaquetas, os biomarcadores circulantes oferecem uma janela complementar para os processos biológicos dinâmicos que sustentam a doença cardiovascular. O perfil seriado de microRNAs (miRNAs) circulantes em pacientes com síndromes coronarianas agudas sem supradesnivelamento do segmento ST (NSTE-ACS) revelou associações genômicas com a reatividade plaquetária.^5^ Em uma análise em duas etapas envolvendo a coorte multiétnica do estudo TRILOGY-ACS e uma coorte asiática independente, miRNAs específicos – especialmente miR-15b-5p, miR-93 e miR-126 – foram consistentemente associados à reatividade plaquetária. Esses achados sugerem que miRNAs circulantes podem refletir mudanças dinâmicas na biologia plaquetária após um evento isquêmico, abrindo caminho para a estratificação da resposta à terapia antiplaquetária guiada por biomarcadores.^5^

Complementando esses *insights* funcionais e transcriptômicos, a lipidômica surgiu como uma ferramenta poderosa para fenotipar a vulnerabilidade da placa aterosclerótica e prever eventos coronarianos agudos. As ceramidas plasmáticas, uma classe de esfingolipídios formados pela condensação de esfingosina e ácidos graxos, participam de múltiplas vias celulares que promovem diretamente a progressão da aterosclerose, incluindo disfunção endotelial, inflamação vascular, infiltração e oxidação de LDL e apoptose de células vasculares. Aproximadamente metade dos pacientes com doença arterial coronariana documentada angiograficamente desenvolve doença multivascular progressiva com altas taxas de mortalidade, mas a prática clínica atual não consegue prever de forma confiável quais pacientes terão eventos isquêmicos recorrentes ou identificar biomarcadores que indiquem claramente instabilidade de placa.

Espécies específicas de ceramidas – particularmente C16:0, C18:0 e C24:1 – foram independentemente associadas ao aumento do risco de eventos cardiovasculares maiores em pacientes com síndrome coronariana aguda. Além disso, imagens por tomografia de coerência óptica demonstraram que essas espécies de ceramidas estavam significativamente elevadas em pacientes com infarto agudo do miocárdio que apresentavam ruptura de placa.^6^ Essa associação independente entre a concentração plasmática de ceramidas e a ruptura de placa representa a primeira evidência direta ligando um biomarcador lipidômico circulante ao substrato patológico dos eventos coronarianos agudos, posicionando as ceramidas como potenciais marcadores de instabilidade e vulnerabilidade da placa aterosclerótica.^6^

Criticamente, a relevância clínica das ceramidas vai além da biologia macrovascular da placa e abrange a dimensão microvascular da doença cardiovascular. Em um estudo multicêntrico prospectivo envolvendo 309 participantes (51 controles saudáveis, 150 pacientes diabéticos sem infarto do miocárdio e 108 pacientes diabéticos com IAM prévio), demonstramos que o mesmo painel de 11 ceramidas plasmáticas melhorou significativamente a predição tanto da doença microvascular coronariana (DMC) quanto da doença microvascular retiniana (DMR), em comparação com razões de ceramidas de referência.^6^ Utilizando análise de curva ROC, o painel de 11 ceramidas alcançou valores de área sob a curva de 0.81 para predição de CMD (p<0.001), 0.73 para predição de RMD (p=0.010) e 0.83 para o desfecho combinado DMC/DMR (p<0.001).

Esses achados sugerem que assinaturas de ceramidas capturam o substrato molecular da disfunção microvascular difusa, refletindo lipotoxicidade, disfunção mitocondrial e estresse metabólico que comprometem simultaneamente as vias ateroscleróticas epicárdicas e a função microcirculatória. Isso é particularmente relevante em populações com diabetes e infarto do miocárdio prévio, nas quais o risco isquêmico residual não é totalmente explicado apenas pela doença coronariana obstrutiva. A integração de biomarcadores moleculares escaláveis na prática clínica exemplifica como a medicina de precisão pode aprimorar a acurácia diagnóstica e a estratificação de risco além dos parâmetros clínicos tradicionais, permitindo identificar pacientes com maior risco tanto de complicações trombóticas agudas quanto de doença microvascular progressiva.

### Escore de Risco Poligênico: Conectando precisão genética e precisão populacional

Um componente transformador da medicina de precisão populacional é a integração dos escores de risco poligênico (ERP), que agregam os efeitos de milhares de variantes genéticas para quantificar o risco cardiovascular hereditário. O UK Biobank, que inclui mais de 500 000 participantes com dados genéticos extensivos, tornou-se um pilar fundamental para o desenvolvimento e validação de ERP para doenças cardiovasculares.^7^ Em um estudo marcante envolvendo 306 654 participantes do UK Biobank sem doença cardiovascular prévia, a adição de um PRS aos fatores de risco convencionais (idade, sexo, pressão arterial sistólica, tabagismo, histórico de diabetes e níveis lipídicos) aumentou o C-index para discriminação de risco cardiovascular de 0,710 para 0,722 — representando uma melhora modesta, porém clinicamente relevante.^7^ Mais importante, o PRS alcançou melhorias contínuas de reclassificação líquida de aproximadamente 10% nos casos e 12% nos não casos, com o maior benefício observado entre indivíduos de risco intermediário (risco cardiovascular de 5% a <10% em 10 anos). Nessa população de risco intermediário, a avaliação direcionada por PRS poderia prevenir um evento cardiovascular adicional para aproximadamente cada 340 indivíduos avaliados — um benefício 1,5 vez maior do que o obtido pela avaliação da proteína C-reativa, um biomarcador incluído em muitas diretrizes de predição de risco.^7^

Entretanto, uma limitação crítica dos primeiros PRS era o desempenho reduzido em indivíduos de ancestralidade não europeia, devido principalmente à sub-representação de populações diversas nos estudos de associação genômica ampla (GWAS). Isso representa outra lacuna importante de dados na medicina de precisão. Para enfrentar esse desafio, esforços recentes têm se concentrado no desenvolvimento de escores poligênicos multiancestrais que integram dados de GWAS de populações diversas. Um escore poligênico multiancestral para doença arterial coronariana (GPS Mult), desenvolvido com dados de mais de 269 000 casos de DAC e 1 178 000 controles de cinco ancestralidades, demonstrou desempenho significativamente aprimorado em todos os grupos de ancestralidade.^8^ Em conjuntos de validação externos incluindo 33.096 participantes africanos, 124 467 europeus, 16 433 hispânicos e 16.874 sul-asiáticos, o GPS Mult superou todos os escores poligênicos de DAC previamente publicados baseados em uma única ancestralidade.

Essa abordagem multiancestral é particularmente importante para pacientes jovens com IAM, que frequentemente são sub-representados nos modelos tradicionais de predição de risco. Ao integrar informações de risco genético com análises estratificadas por idade, os PRS podem identificar indivíduos jovens de alto risco que seriam ignorados pelos escores clínicos convencionais.^8^

### Implementação da Medicina de Precisão Baseada em Sistemas

A concretização da medicina de precisão populacional exige mais do que novos biomarcadores e escores de risco recalibrados; ela requer sistemas de saúde robustos e interconectados, capazes de oferecer cuidado direcionado. O ensaio clínico randomizado IMMACULATE demonstrou a viabilidade de uma abordagem baseada em sistemas ao avaliar um programa de manejo intensivo remoto, habilitado por telemedicina e conduzido por enfermeiros especialistas, para pacientes com IAM após a alta hospitalar.^9^ Embora essa intervenção específica, aplicada a uma coorte de baixo risco, tenha demonstrado segurança e viabilidade comparáveis ao cuidado liderado por cardiologistas, ela destaca o potencial de utilizar tecnologia e profissionais de saúde aliados para implementar acompanhamento de precisão e entrega contínua de cuidados adaptados ao risco.^9^

Da mesma forma, a implementação inicial de ERP na prática clínica já está em andamento. Em 2024, doze unidades de atenção primária no Norte da Inglaterra testaram a integração de ERP com o QRISK, um escore convencional de risco cardiovascular, para orientar decisões sobre terapia com estatinas. Médicos generalistas relataram que o conhecimento do ERP mudaria seu plano de tratamento em 13% dos casos, e os pacientes consideraram úteis as discussões sobre seu risco genético, com melhora na adesão a medicamentos preventivos e modificações de estilo de vida. Esses achados demonstram que PRS pode ser incorporado à prática rotineira da atenção primária para aprimorar a estratificação de risco e apoiar a tomada de decisão clínica.^7^

### Rumo a uma medicina de precisão populacional integrada: Além do paradigma “Um Paciente, Um Fármaco”

É importante enfatizar que a medicina de precisão populacional em cardiologia não exige necessariamente a descoberta de um fármaco único para cada paciente ou grupo étnico. Em vez disso, ela demanda um equilíbrio mais sofisticado das estratégias terapêuticas já existentes – guiado por biomarcadores e por uma estratificação de risco refinada – para otimizar as taxas de sucesso em populações diversas. Por exemplo, reconhecer que pacientes malaios apresentam maior mortalidade apesar de escores GRACE mais baixos não exige um novo agente terapêutico; exige, sim, a recalibração da avaliação de risco e a intensificação de intervenções comprovadas para esse fenótipo de alto risco.

Da mesma forma, identificar alta reatividade plaquetária não implica a necessidade de desenvolver um novo antiplaquetário. Em vez disso, orienta a otimização das estratégias antiplaquetárias existentes — incluindo ajustes de dose, escolha do antagonista P2Y12 mais adequado e individualização da duração da terapia antiplaquetária dupla. A integração do perfil de ceramidas com a imagem microvascular não introduz novos medicamentos, mas permite que os clínicos reconheçam pacientes com disfunção microvascular difusa que se beneficiariam de controle metabólico mais agressivo, manejo intensivo de lipídios e vigilância clínica ampliada.

Nesse paradigma, a medicina de precisão torna-se um framework para a alocação inteligente dos recursos terapêuticos existentes – garantindo que a intensidade correta da intervenção chegue ao paciente certo no momento certo.

À medida que avançamos na ciência cardiovascular, a transição da medicina estratificada – que agrupa pacientes por perfis amplos de risco – para uma verdadeira medicina de precisão dependerá da nossa capacidade de integrar múltiplas camadas de informação: fatores étnicos e demográficos, fatores clínicos convencionais de risco, fenótipos funcionais dinâmicos (como a reatividade plaquetária), biomarcadores moleculares dinâmicos (incluindo miRNAs e assinaturas lipidômicas) e risco genético hereditário. Isso exige um esforço coordenado para validar biomarcadores e escores de risco em diversos grupos étnicos, como demonstrado por pesquisas colaborativas que vão de Singapura ao Brasil, e para adaptar nossos sistemas de prestação de cuidados de saúde de acordo com essas necessidades.

As lacunas de dados identificadas – disparidades étnicas no desempenho de escores de risco, mortalidade oculta entre sobreviventes jovens de IAM, desempenho reduzido de PRS em populações de ancestralidade não europeia e a incapacidade de prever instabilidade de placa usando apenas parâmetros clínicos convencionais – apontam para a mesma necessidade fundamental: a medicina de precisão populacional deve ser inclusiva, multiétnica e continuamente refinada à medida que novos dados surgem. O UK Biobank e outros biobancos de grande escala e diversidade representam uma infraestrutura essencial para esse objetivo, permitindo o desenvolvimento de modelos de risco que funcionem de maneira equitativa entre diferentes populações.

## Conclusão

A medicina de precisão populacional em cardiologia representa uma evolução necessária diante das limitações do modelo do “paciente médio”. Ao incorporar diversidade étnica na estratificação de risco, integrar biomarcadores funcionais dinâmicos como fenótipos de receptores plaquetários, incluir biomarcadores moleculares como miRNAs e ceramidas que refletem diretamente a vulnerabilidade da placa, adotar escores de risco poligênico desenvolvidos e validados em populações diversas e inovar na prestação de cuidados por meio de sistemas de saúde conectados, podemos cumprir a promessa de oferecer o cuidado cardiovascular certo ao paciente certo no momento certo. As evidências mostram cada vez mais que abordagens convencionais de “tamanho único” falham em capturar a complexidade dos diferentes fenótipos de pacientes, e que a medicina de precisão – fundamentada em dados específicos de cada população, validação multiétnica e biomarcadores com base mecanística – oferece um caminho para um cuidado cardiovascular mais equitativo e eficaz globalmente.

## References

[1] Carvalho L. P (2021). Precision Medicine in Singapore: What it's Worth for?.

[2] Carvalho LP, Gao F, Chen Q, Hartman M, Sim LL, Koh TH (2014). Differences in Late Cardiovascular Mortality Following Acute Myocardial Infarction in Three Major Asian Ethnic Groups. Eur Heart J Acute Cardiovasc Care.

[3] Jing M, Gao F, Chen Q, Carvalho LP, Sim LL, Koh TH (2016). Comparison of Long-Term Mortality of Patients Aged ≤40 versus >40 Years with Acute Myocardial Infarction. Am J Cardiol.

[4] Carvalho LP, Fong A, Troughton R, Yan BP, Chin CT, Poh SC (2018). Prognostic Implications of Dual Platelet Reactivity Testing in Acute Coronary Syndrome. Thromb Haemost.

[5] Becker KC, Kwee LC, Neely ML, Grass E, Jakubowski JA, Fox KAA (2020). Circulating MicroRNA Profiling in Non-ST Elevated Coronary Artery Syndrome Highlights Genomic Associations with Serial Platelet Reactivity Measurements. Sci Rep.

[6] Junqueira DL, Cavalcanti AB, Sallum JMF, Yasaki E, Jesuíno IA, Stach A (2024). Plasma Ceramides as Biomarkers for Microvascular Disease and Clinical Outcomes in Diabetes and Myocardial Infarction. Clin Diabetes Endocrinol.

[7] Sun L, Pennells L, Kaptoge S, Nelson CP, Ritchie SC, Abraham G (2021). Polygenic Risk Scores in Cardiovascular Risk Prediction: A Cohort Study and Modelling Analyses. PLoS Med.

[8] Patel AP, Wang M, Ruan Y, Koyama S, Clarke SL, Yang X (2023). A Multi-Ancestry Polygenic Risk Score Improves Risk Prediction for Coronary Artery Disease. Nat Med.

[9] Chan MY, Koh KWL, Poh SC, Marchesseau S, Singh D, Han Y (2021). Remote Postdischarge Treatment of Patients with Acute Myocardial Infarction by Allied Health Care Practitioners vs Standard Care: The IMMACULATE Randomized Clinical Trial. JAMA Cardiol.

