# Supplementary material for: Medicina de Precisão Populacional em Cardiologia: Reduzindo Lacunas em Saúde por Meio da Estratificação Integrada de Risco
Source: Arq Bras Cardiol. 2026 Jul 31;123(7):e20260546. [Article in Portuguese] doi: 10.36660/abc.20260546 (PMC13668451; doi:10.36660/abc.20260546)
Supplement: Translation

# Translation: Population Precision Medicine in Cardiology: Bridging Health Gaps Through Integrated Risk Stratification

**DOI:** 10.36660/abc.20260546i

**Authors:** Leonardo Pinto de Carvalho, Mark Y. Chan

**Affiliations:** 1 Brazil Disciplina de Cardiologia – Escola Paulista de Medicina – Universidade Federal de São Paulo, São Paulo, SP – Brazil; 2 Brazil Dante Pazzanese Institute of Cardiology (IDPC), São Paulo, SP – Brazil; 3 Singapore Departamento de Cardiologia – Yong Loo Lin School of Medicine – National University of Singapore – Singapore; 4 Singapore Cardiovascular Disease National Collaborative Enterprise (CADENCE), Rochester Park – Singapore

**Keywords:** Population Precision Medicine, Integrated Risk Stratification, Cardiology

## Introduction

The paradigm of cardiovascular medicine is undergoing a profound transformation. For decades, the cornerstone of evidence-based cardiology has been the application of findings from large randomized clinical trials to individual patients. While this “one-size-fits-all” approach has undeniably reduced cardiovascular morbidity and mortality worldwide, it inherently assumes that the “average” patient represented in clinical trials accurately reflects the individual sitting in the consultation room. In practice, clinicians continue to struggle with substantial patient variability, delayed or imprecise diagnosis of both rare and common diseases, and treatments designed for the average patient often show limited effectiveness when applied across diverse populations.^1^

We are now entering the era of precision medicine, which promises to replace imprecise care with data-driven strategies that integrate population-level information with molecular insights. This approach aims to generate targeted treatment and care plans based on each patient’s unique clinical, genomic, and lifestyle characteristics.

In cardiology, however, precision medicine must extend beyond individual genetic sequencing to encompass population precision medicine. This broader perspective recognizes that ethnic, demographic, systemic, and genetic factors profoundly influence cardiovascular risk, disease phenotypes, and treatment responses. The widespread use of conventional risk scores across multiethnic populations – combined with emerging evidence of hidden mortality risks in specific age groups, the integration of dynamic molecular biomarkers, and the incorporation of polygenic risk scores – highlights the urgent need for this paradigm shift.

### Ethnic disparities and the limitations of conventional risk scores

A compelling example of the need for population-specific precision medicine is illustrated by the long-term outcomes following acute myocardial infarction (AMI). In a study evaluating more than 15,000 AMI patients across three major Asian ethnic groups (Chinese, Malay, and Indian) in Singapore, significant disparities in long-term mortality were identified.^2^ Although Chinese patients presented with the highest baseline risk according to the Global Registry of Acute Coronary Events (GRACE) score—with a median score of 144 compared with 138 in Malay and 131 in Indian patients—the 12-year cardiovascular mortality pattern differed markedly.

Malay patients exhibited the highest 12-year overall and cause-specific cardiovascular mortality (46.2% and 32.0%, respectively), followed by Chinese (43.0% and 27.0%) and Indian (35.9% and 25.2%) patients. This discordance between conventional baseline risk stratification and long-term outcomes highlights a critical limitation: risk scores derived predominantly from specific populations may not accurately translate to others.

Therefore, population precision medicine requires the recalibration of established risk models to account for ethnic heterogeneity, ensuring that high-risk subgroups receive the intensified interventions they truly need.^2^

### Hidden mortality in young patients: Unmasking data gaps

Another critical data gap in precision cardiovascular medicine concerns young patients with AMI. Despite the conventional belief that young AMI patients have a more favorable prognosis than older individuals, a comprehensive analysis of young AMI patients in Singapore revealed a sobering reality when these patients were compared with age-matched background population controls rather than older MI patients.^3^ Among 601 young patients aged ≤40 years, the 12-year all-cause and cardiovascular mortality rates were 12.8% and 9.2%, respectively – fivefold lower than in older patients. However, when compared with an age-matched background population using the relative survival ratio (RSR) methodology, young AMI patients remained at significantly elevated risk.

The five-year RSR for young patients was 0.942 (95% CI 0.918–0.960), indicating that their survival was approximately 5.8% worse than expected for their age group. This finding highlights a critical insight: young AMI survivors are “flying under the radar” of conventional risk assessment. They appear to have favorable outcomes when compared only with older MI patients, yet they carry substantial excess mortality relative to their age-matched peers who have not experienced MI.

This discordance represents a major gap in cardiovascular care – the failure to recognize that young AMI patients constitute a distinct and complex clinical phenotype that is not accurately captured by common risk factors. Their evaluation requires specialized molecular and genetic testing to ensure proper diagnosis and risk stratification.^3^

### Molecular biomarkers and dynamic risk stratification: From platelet receptor phenotypes to lipidomic signatures

The evolution toward precision medicine is increasingly supported by advances in molecular phenotyping and omics technologies. Understanding the acute phase of myocardial infarction requires an appreciation of the dynamic interplay among multiple biological systems, beginning with the functional phenotypes of platelet receptor–mediated responses. Notably, even before circulating microRNA profiling emerged as a candidate precision tool, functional studies had already demonstrated that early modulation of platelet receptor–mediated reactivity during acute coronary syndromes carries prognostic significance.

In a prospective study of 928 consecutive patients hospitalized for acute coronary syndrome, dual platelet reactivity testing revealed that high on-aspirin platelet reactivity (HASPR) and high on-adenosine diphosphate receptor antagonist platelet reactivity (HADPR) were independently associated with increased in-hospital stent thrombosis and major adverse cardiovascular events (MACE).^4^ Importantly, the addition of initial HADPR measurements to the GRACE score significantly improved discrimination for 30-day MACE (C-statistic increased from 0.649 to 0.803), demonstrating that functional platelet receptor phenotypes provide incremental prognostic information beyond conventional clinical risk stratification. This suggests that receptor-driven platelet phenotypes in the acute phase are not merely epiphenomena but mechanistically relevant determinants of thrombotic risk that warrant incorporation into precision medicine frameworks.

Building on this foundation of functional platelet biology, circulating biomarkers offer a complementary window into the dynamic biological processes underlying cardiovascular disease. Serial profiling of circulating microRNAs (miRNAs) in patients with non–ST-segment elevation acute coronary syndromes (NSTE-ACS) has revealed genomic associations with platelet reactivity.^5^ In a two-stage analysis encompassing the multiethnic TRILOGY-ACS trial cohort and an independent Asian cohort, specific miRNAs – most notably miR-15b-5p, miR-93, and miR-126 – were consistently associated with platelet reactivity. These findings suggest that circulating miRNAs can report on dynamic changes in platelet biology following an ischemic event, paving the way for biomarker-driven stratification of antiplatelet therapy response.^5^

Complementing these functional and transcriptomic insights, lipidomics has emerged as a powerful tool for phenotyping atherosclerotic plaque vulnerability and predicting acute coronary events. Plasma ceramides, a class of sphingolipids formed through the condensation of sphingosine and fatty acids, participate in multiple cellular pathways that directly promote atherosclerotic progression, including endothelial dysfunction, vascular inflammation, LDL infiltration and oxidation, and apoptosis of vascular cells. Approximately half of patients with angiographically documented coronary artery disease develop progressive multivessel disease with high mortality rates, yet current clinical practice cannot reliably predict which patients will experience recurrent ischemic events or identify biomarkers that clearly indicate plaque instability.

Specific ceramide species – particularly C16:0, C18:0, and C24:1 – have been independently associated with increased risk of major adverse cardiovascular events in patients with acute coronary syndrome. Moreover, optical coherence tomography imaging demonstrated that these ceramide species were significantly elevated in patients with acute myocardial infarction who exhibited plaque rupture.^6^ This independent association between plasma ceramide concentration and plaque rupture represents the first direct evidence linking a circulating lipidomic biomarker to the pathological substrate of acute coronary events, positioning ceramides as candidate markers of atherosclerotic plaque instability and vulnerability.^6^

Critically, the clinical relevance of ceramides extends beyond macrovascular plaque biology and encompasses the microvascular dimension of cardiovascular disease. In a prospective multicenter study enrolling 309 participants (51 healthy controls, 150 diabetic patients without myocardial infarction, and 108 diabetic patients with prior AMI), we demonstrated that the same panel of 11 plasma ceramides significantly improved prediction of both coronary microvascular disease (CMD) and retinal microvascular disease (RMD), compared with reference ceramide ratios.^6^ Using receiver operating characteristic curve analysis, the 11-ceramide panel achieved area-under-the-curve values of 0.81 for CMD prediction (p<0.001), 0.73 for RMD prediction (p=0.010), and 0.83 for the combined CMD/RMD outcome (p<0.001).

These findings suggest that ceramide signatures capture the molecular substrate of diffuse microvascular dysfunction, reflecting lipotoxicity, mitochondrial dysfunction, and metabolic stress that simultaneously compromise both epicardial atherosclerotic pathways and microcirculatory function. This is particularly relevant in populations with diabetes and prior myocardial infarction, in whom residual ischemic risk is not fully explained by obstructive coronary disease alone. The integration of such scalable molecular biomarkers into clinical practice exemplifies how precision medicine can enhance diagnostic accuracy and risk stratification beyond traditional clinical parameters, enabling identification of patients at highest risk for both acute thrombotic complications and progressive microvascular disease.

### Polygenic Risk Scores: Bridging genetic and population precision

A transformative approach to population precision medicine is the integration of polygenic risk scores (PRS), which aggregate the effects of thousands of genetic variants to quantify inherited cardiovascular risk. The UK Biobank, comprising more than 500,000 participants with extensive genetic data, has become a cornerstone for developing and validating PRS for cardiovascular disease.^7^ In a landmark study of 306,654 UK Biobank participants without prior cardiovascular disease, adding a PRS to conventional risk factors (age, sex, systolic blood pressure, smoking status, diabetes history, and lipid levels) increased the C-index for CVD risk discrimination from 0.710 to 0.722 – representing a modest but clinically meaningful improvement.^7^ More importantly, the PRS achieved continuous net reclassification improvements of approximately 10% in cases and 12% in non-cases, with the greatest benefit observed among individuals at intermediate risk (5% to <10% 10-year CVD risk). In this intermediate-risk population, targeted PRS assessment could prevent one additional CVD event for approximately every 340 individuals screened – a 1.5-fold greater benefit than C-reactive protein assessment, a biomarker included in many risk prediction guidelines.^7^

However, a critical limitation of early PRS development was their reduced performance in individuals of non-European ancestry, largely due to the underrepresentation of diverse populations in genome-wide association study (GWAS) discovery cohorts. This represents another important data gap in precision medicine. To address this, recent efforts have focused on developing multi-ancestry polygenic scores that integrate GWAS data from diverse populations. A multi-ancestry polygenic score for coronary artery disease (GPS Mult), developed using data from more than 269,000 CAD cases and 1,178,000 controls across five ancestries, demonstrated significantly improved performance across all ancestry groups.^8^ In external validation datasets including 33,096 African, 124,467 European, 16,433 Hispanic, and 16,874 South Asian participants, GPS Mult outperformed all previously published single-ancestry CAD polygenic scores.

This multi-ancestry approach is particularly important for young AMI patients, who are often underrepresented in traditional risk prediction models. By integrating genetic risk information with age-stratified analysis, PRS can identify high-risk young individuals who would otherwise be missed by conventional clinical risk scores.^8^

### Systems-based implementation of precision medicine

The realization of population precision medicine requires more than novel biomarkers and recalibrated risk scores; it demands robust, interconnected healthcare systems capable of delivering targeted care. The IMMACULATE randomized clinical trial illustrated the feasibility of a systems-based approach by evaluating a telehealth-enabled, nurse practitioner–led remote intensive management program for post-discharge AMI patients.^9^ Although this specific intervention in a low-risk cohort demonstrated safety and feasibility comparable to standard cardiologist-led care, it highlights the potential of leveraging technology and allied healthcare professionals to implement precision follow-up and continuous, risk-adapted care delivery.^9^

Similarly, early implementation of PRS in clinical practice is already underway. In 2024, twelve general practices in the North of England tested the integration of PRS with QRISK, a conventional cardiovascular risk score, to guide statin therapy decisions. General practitioners reported that knowledge of the PRS would change their treatment plan in 13% of cases, and patients found discussions about their genetic risk helpful, with improved uptake of preventive medications and lifestyle modifications. These findings demonstrate that PRS can be incorporated into routine primary care to enhance risk stratification and support clinical decision-making.^7^

### Toward integrated population precision medicine: Beyond the “One Patient, One Drug” paradigm

It is important to emphasize that population precision medicine in cardiology does not require discovering a unique drug for each patient or ethnic group. Rather, it demands a more sophisticated balancing of existing therapeutic strategies – guided by biomarkers and refined risk stratification – to optimize treatment effectiveness across diverse populations. For example, recognizing that Malay patients experience higher mortality despite lower GRACE scores does not necessitate a novel therapeutic agent; instead, it calls for recalibrated risk assessment and intensification of proven interventions tailored to this high-risk phenotype.

Similarly, identifying high platelet reactivity does not mandate the development of a new antiplatelet drug. Instead, it informs the optimization of current antiplatelet strategies, including dosing adjustments, selection of the most appropriate P2Y12 antagonist, and individualized duration of dual antiplatelet therapy. The integration of ceramide profiling with microvascular imaging does not introduce new pharmacological treatments, but it enables clinicians to identify patients with diffuse microvascular dysfunction who may benefit from more aggressive metabolic control, intensified lipid management, and enhanced clinical surveillance.

Within this paradigm, precision medicine becomes a framework for the intelligent allocation of existing therapeutic resources – ensuring that the right intensity of intervention reaches the right patient at the right time.

As cardiovascular science advances, the transition from stratified medicine – grouping patients by broad risk profiles – to true precision medicine will depend on our ability to integrate multiple layers of information: ethnic and demographic factors, conventional clinical risk variables, dynamic functional phenotypes (such as platelet reactivity), dynamic molecular biomarkers (including miRNAs and lipidomic signatures), and inherited genetic risk. Achieving this requires a coordinated effort to validate biomarkers and risk scores across diverse ethnic groups, as demonstrated by collaborative research spanning regions from Singapore to Brazil, and to adapt healthcare delivery systems accordingly.

The data gaps identified – ethnic disparities in risk score performance, hidden mortality among young AMI survivors, reduced PRS performance in individuals of non-European ancestry, and the inability of conventional clinical parameters to predict plaque instability – converge on a single fundamental need: population precision medicine must be inclusive, multiethnic, and continuously refined as new evidence emerges. The UK Biobank and other large-scale, diverse biobanks represent essential infrastructure for this endeavor, enabling the development of risk models that perform equitably across populations.

## Conclusion

Population precision medicine in cardiology represents a necessary evolution beyond the limitations of the “average patient” paradigm. By embracing ethnic diversity in risk stratification, integrating dynamic functional biomarkers such as platelet receptor phenotypes, incorporating molecular biomarkers like miRNAs and ceramides that directly reflect plaque vulnerability, adopting polygenic risk scores developed and validated in diverse populations, and innovating care delivery through connected health systems, we can fulfill the promise of delivering the right cardiovascular care to the right patient at the right time. Growing evidence demonstrates that conventional one-size-fits-all approaches fail to capture the complexity of distinct patient phenotypes, and that precision medicine – rooted in population-specific data, multiethnic validation, and mechanistically grounded biomarkers – offers a pathway toward more equitable and effective cardiovascular care worldwide.
